# Evaluation of endothelialization of an occluder device with cardiac computed tomography and assessment of the pathological validation

**DOI:** 10.1371/journal.pone.0316638

**Published:** 2025-01-10

**Authors:** Ang Liu, Xuejing Duan, Ke Wang, Hongguang Fan, Li Li, Chaowu Yan

**Affiliations:** 1 Department of Structural Heart Disease, Cardiovascular Institute and Fuwai Hospital, National Center for Cardiovascular Diseases, Chinese Academy of Medical Sciences and Peking Union Medical College, Beijing, China; 2 Department of Pathology, Cardiovascular Institute and Fuwai Hospital, Beijing, China; 3 Department of Surgery, Cardiovascular Institute and Fuwai Hospital, Beijing, China; Al Nasiriyah Teaching Hospital, IRAQ

## Abstract

**Background:**

Assessing the endothelialization of occlusive devices noninvasively remains a challenge. Cardiac computed tomography angiography (CTA) can be employed to evaluate device endothelialization based on contrast uptake within the occluder.

**Objective:**

This study examined device endothelialization using cardiac CTA and investigated the pathological associations.

**Methods:**

From January 2010 to May 2022, we retrospectively analyzed 25 patients (age: 50.00 [17.00, 52.00] years; 12 Female) who underwent surgical device removal within 1 month after cardiac CTA examination (implantation period: 29.00[0.50, 108.00] months). The contrast uptake within the occluder was determined using cardiac CTA. The relationship between contrast uptake within the occluder and the endothelialization status with pathology was analyzed.

**Results:**

Contrast uptake within the occluder was identified in 76.00% of patients. Pathological examination confirmed incomplete coverage of fibrotic tissue and superposed neoendothelium on the surface of all devices exhibiting contrast uptake. This included no coverage in 47.37% of patients and partial coverage in the remaining cases. On the surface of all devices without contrast uptake, a complete range of fibrotic tissue was observed, with an incomplete range of superposed neoendothelium in 66.67% of patients. On the surface of devices with an implantation period > 6 months, 71.43% of patients had incomplete coverage of fibrotic tissue and superposed neoendothelium on the left disc, 42.86% of patients occurred the same on the right disc.

**Conclusions:**

Contrast uptake within the occluder indicated incomplete endothelialization, as confirmed by pathological validation. Late endothelialization of the device occurs frequently, and further research is required to investigate related mechanisms.

## Introduction

With the advent of nitinol mesh occluders, transcatheter closure has become the preferred strategy for a variety of cardiovascular diseases, such as atrial septal defects, patent ductus arteriosus, ventricular septal defects, patent foramen ovale, and perivalvular leakage [1–4]. It is generally accepted that an implanted occluder becomes fully endothelialized within 3 months (data from animal studies), which is the basis for determining antiplatelet therapy duration in humans [5, 6]. After transcatheter closure, 6 months of anti-aggregation therapy is recommended to prevent thrombus formation in the device. However, with the worldwide application of device occluders, late device endothelialization has been increasingly reported, with some cases being associated with endocarditis [7–11]. Hence, the duration of antiplatelet therapy and antibiotic prophylaxis following closure is becoming a subject of controversy.

Developing a noninvasive technique to evaluate the endothelialization status of the device is imperative. Previous studies have demonstrated that CTA could serve as a noninvasive assessment method, yet pathological validation remains lacking [12, 13]. Adequate endothelialization of the nitinol mesh occluder results in complete isolation of the device surface from contact with the blood. Otherwise, blood can freely enter the occluder through potential contact with surfaces with insufficient endothelialization. Theoretically, the presence of contrast uptake within the occluder on cardiac CTA will reflect insufficient endothelization of the device [12, 14]. Therefore, we hypothesized that cardiac CTA could be used to evaluate the endothelialization status of devices in humans. This study aimed to evaluate the association between cardiac CTA findings and pathological examination in the assessment of device endothelialization.

## Methods

### Study design and participants

This retrospective study was conducted between January 2010 and May 2022 at Fuwai Hospital (Beijing, China). The clinical data were accessed and extracted for research purposes on 29/12/2022. The inclusion criteria were as follows: (1) patients who underwent cardiac surgery for conditions including, but not limited to, device migration, atrioventricular valve disease, or aortic valve disease; (2) cardiac occluders were removed during the surgery. The exclusion criteria encompassed patients who were unable to undergo cardiac CTA examination due to various contraindications, including but not limited to history of allergic reactions to iodinated contrast media. The Ethics Committee of Fuwai Hospital approved this study, and written informed consent was obtained from each patient. All personal information was anonymized and de-identified prior to analysis.

In total, 25 consecutive candidates underwent surgical device removal, and all patients underwent preoperative cardiac CTA examination within 1 month. The study included 16 patients with an atrial septal occluder, 5 with a patent ductus occluder, 2 with a ventricular septal occluder, 1 with a vascular occluder and 1 with a left atrial appendage occluder. Furthermore, two types of nitinol mesh occluders with similar designs were included: 1) amplatzer occluders (n = 16) and 2) ceramic occluders (n = 9).

In this study, preoperative cardiac CTA was performed for surgical planning. The presence or absence of contrast uptake within the occluder was determined using cardiac CTA. Within 1 month of the cardiac CTA examination, the device was surgically removed and analyzed pathologically. Furthermore, contrast uptake within the occluder and pathological findings were compared. The implantation period was defined as the time between device implantation and surgical removal.

### Cardiac CTA examination

Electrocardiography-gated contrast-enhanced cardiac CTA was performed to assess the implanted devices [15, 16]. CTA was performed with two CT scanners: a third-generation dual-source CT scanner (SOMATOM Force; Siemens Heatlhineers, Germany), and a 256-section wide-detector CT scanner (Revolution HD; GE Healthcare, U.S.). A 100-kV tube potential was used for patients with a body mass index (BMI) < 30 kg/m^2^, while a 120-kV tube potential was used for those with a BMI ≥ 30 kg/m^2^. The X-ray tube current was adjusted individually for each patient according to their BMI. To enhance the contrast medium, automated bolus tracking was used in a region of interest (ROI) within the ascending aorta (signal attenuation trigger threshold of 100 Hounsfield units [HU], 6-s scan delay). The leading scanning parameters were an individual detector width of 0.6 mm, a gantry rotation time of 280 ms, a pitch of 0.20–0.50, and a field of view of 200–250 mm for raw image reconstruction. A triple-phase contrast medium injection protocol was used, which consisted of 50–60 mL of undiluted contrast agent (iopromide, 370 mg/mL; Bayer Healthcare, Berlin, Germany), followed by a 30-mL 30%:70% mixture of contrast medium and saline, along with a 30-mL saline chaser bolus, all injected at flow rates of 4–5 mL/s. After the examination, 10 phases were reconstructed throughout the cardiac cycle, with the RR interval divided into 10% increments.

During end systole, the in vivo device size (waist size of the occluder on CTA images) and device thickness (distance between the two discs at the mid-portion) were measured in planes that cut through the two metallic points on each side of the device. The local CT density was determined within the device, myocardium (mid-portion of the muscular interventricular septum), and left atrium. Contrast uptake within the occluder was defined as when the local CT density of the ROI within the device being higher than that of the myocardium (mid-portion of the muscular interventricular septum). Contrast enhancement in the entire device was considered complete uptake, while any visible diffusions of contrast through the part of the discs were classified as partial uptake. If there was no contrast within the device and the shape of the device was flattened, that was defined no contrast uptake. The images were independently assessed by two independent experienced radiologists (with more than 10 years of experience in cardiovascular imaging) blinded to the patient’s profile and implanted device information, and a consensus was reached.

### Pathology

After surgical removal of the implanted device, the occluder was examined grossly. After entering the central hub, the device surface was divided into four quadrants. The device was fixed in a 10% formalin solution and two tissues from each quadrant were taken and one section was made from each specimen. The sections were staining with H&E, Masson trichrome and immunohistochemical staining of CD31 which was the biomarker of endothelia. Totally, there were 8 sections examined in each occluder. The tissue sections taken from the occluder were observed under light microscopy and the ratio of endotheliazation was calculated by area of CD31 positive endothelia covered on the surface of tissues divided by total surface area. The mean endothelialization rate was calculated for the four quadrants.

Complete endothelialization was defined as full coverage of a smooth layer of neoendothelium on the surface of the device and the absence of thrombi or vegetation adjacent to the surface of the implant. Incomplete endothelialization was defined as partial or no coverage of the neoendothelium on the device. The images were analyzed by two independent pathologists who were blinded to patient information.

### Statistical analysis

Categorical variables were expressed as percentages and proportions, and were tested using the chi-square test, Fisher exact test, or Wilcoxon rank sum test. Continuous variables were expressed as mean (standard deviation [SD]) or median (interquartile range [IQR]) and tested using the t-test or Wilcoxon rank sum test for normally distributed variables and skewed-distributed variables, respectively. Spearman’s correlation analysis was performed to evaluate the associations between endothelialization rates and various factors, including implantation duration, device size, antiplatelet therapy duration, and other relevant parameters. All tests were two-tailed, and P<0.05 was considered statistically significant. All analyses were performed using SPSS 16.0 software.

## Results

### Clinical characteristics

**Table 1** shows the clinical characteristics of the 25 patients included in this study. The mean implantation period was 29.00 [0.50, 108.00] months. The reasons for surgical removal of the occluder included dislodgement of the device in 5 patients, atrioventricular valvuloplasty/repair in 11, aortic repair in 3, aortic valvuloplasty in 2, heart transplantation after left atrial appendage occlusion in 1, hemolysis after ventricular septal defect closure in 1, persistent severe postoperative headache in 1, and atrioventricular block after atrial septal defect closure in 1. Preoperatively, none of the patients experienced symptomatic thromboembolic events or infective endocarditis. After closure, the implant period was > 6 months in 14 patients (56.00%), including 12 patients with atrial or ventricular septal defects who received antiplatelet therapy for 6–12 months. Before surgical removal of the devices, 13 patients underwent anticoagulation therapy.

**Table 1:** Baseline characteristics of the included patients (n = 25).

Characteristics	Total Population (n = 25)
Age (yrs)	50.00 [17.00, 52.00]
Female (%)	12.00 (48.00)
Height (cm)	165.00 [155.00, 170.00]
Weight (kg))	56.84 ±18.06
BMI (kg/ m^2^)	21.71 ±4.08
Devices, n(%)	
Atrial septal occluder	16.00 (64.00)
Ventricular septal occluder	2.00 (8.00)
Patent ductus occluder	5.00 (20.00)
Left atrial appendage occluder	1.00 (4.00)
Vascular occluder	1.00 (4.00)
Device size (mm)	19.56 ± 9.74
Device thickness (mm)	12.02 ±3.77
Implant period (months)	29.00 [0.50,108.00]

Values are median [IQR] (Interquartile range), n (%), or mean ± SD. (BMI = body mass index)

### Cardiac CT–pathological correlation

The in vivo device size was 19.56 ± 9.74 mm, and the in vivo device thickness was 12.02 ± 3.77 mm. Within the occluder, contrast uptake was detected in 76.00% of patients (n = 19; implantation period < 3 months in 9, 3–6 months in 1 and > 6 months in 9), including partial uptake in 10 and complete uptake in 9. Compared to those without contrast uptake, the implantation period tended to be shorter in the devices with contrast uptake (4.00 [0.38, 69.00] months vs. 89.50 [50.75, 126.00] months, P = 0.06).

**Fig 1:**
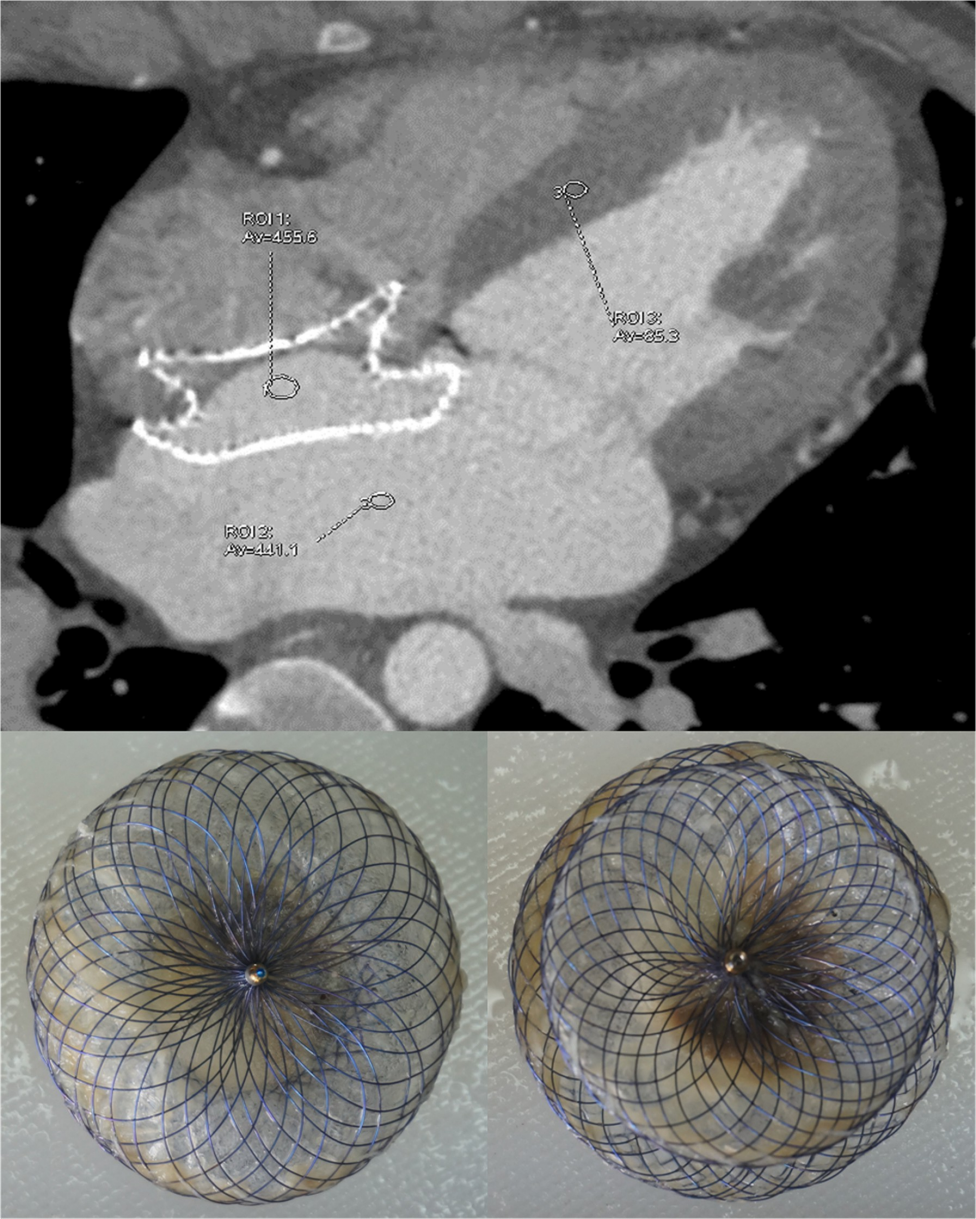
Complete contrast uptake within the occluder on cardiac computed tomography angiography (CTA). Cardiac CTA demonstrated complete contrast uptake within the occluder in a patient with an implantation period of 4 years (upper panel). On either disc, there was almost no endothelialization with complete exposure of device struts (lower). *Note*: *local CT density was 455*.*6 HU within the device*, *85*.*3 HU in the myocardium*, *and 441*.*1 HU in the left atrium; ROI*, *region of interest; Av*, *average CT density; CT*, *computed tomography; HU*, *Hounsfield units*.

**Fig 2:**
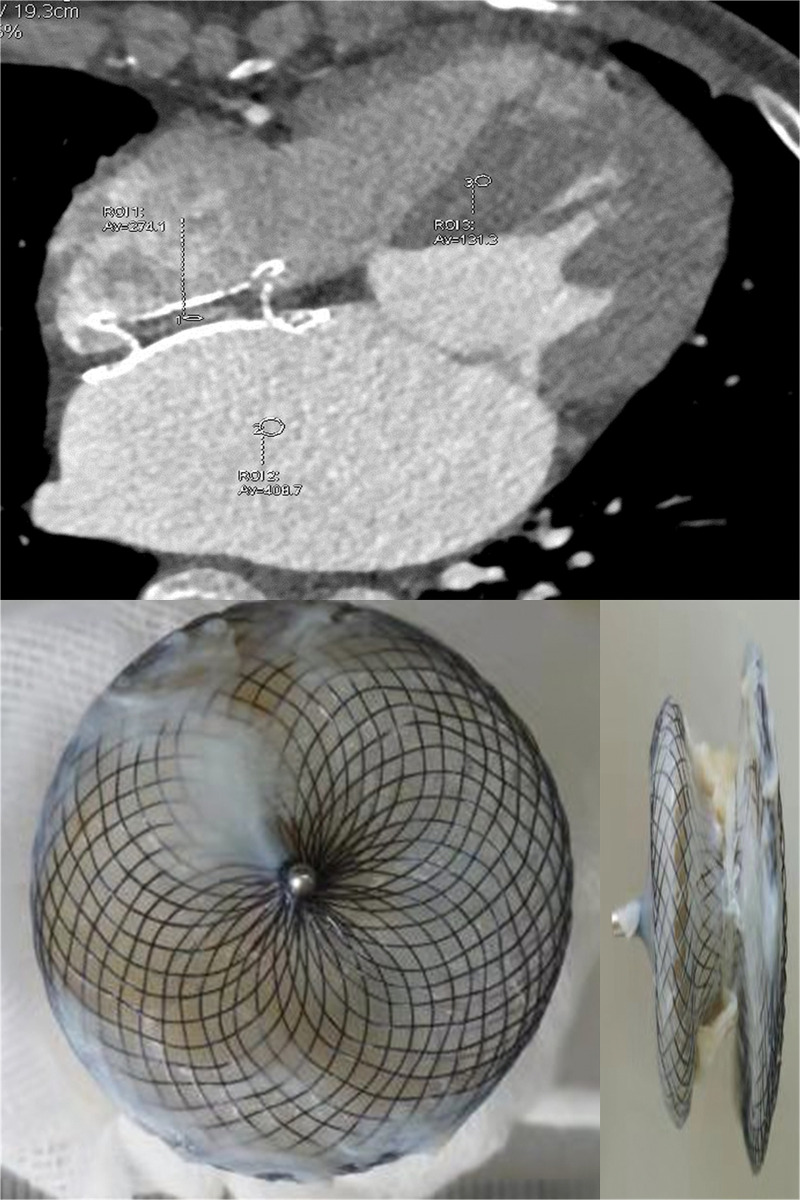
Partial contrast uptake within the occluder on cardiac computed tomography angiography (CTA). Partial contrast uptake was identified within the occluder in a patient with an implantation period of 2 years (upper panel). The local CT density of the contrast uptake area within the device exceeded that of the myocardium, and partial endothelialization was detected on the disc of the device (lower panel). The tissue from the central area (arrows) on the device indicated fibrotic tissue on the partially endothelialized device. *Note*: *local CT density was 274*.*1 HU within the contrast uptake area of the device*, *131*.*3 HU in the myocardium*, *and 408*.*7 HU in the left atrium; ROI*, *region of interest; Av*, *average CT density; CT*, *computed tomography; HU*, *Hounsfield units*.

On the surfaces of all devices with contrast uptake, incomplete coverage of the fibrotic tissue and superposed neoendothelium was confirmed pathologically. The left discs (1.00 [0.00, 2.00] quadrants) and right discs (0.00 [0.00, 3.50] quadrants) were partially covered by fibrotic tissue. Furthermore, in devices with complete contrast uptake (47.37%), histological examination revealed minimal to absent fibrotic tissue and superposed neoendothelium, while the remaining devices demonstrated partial contrast uptake (52.63%), and multiple thrombi were identified both grossly and histologically on the polyester fibers (n = 8).Pathological examination revealed that contrast uptake corresponded to incomplete endothelialization. (**Table 2; Figs 1 and 2**).

**Table 2:** Endothelialization of devices with and without contrast uptake.

	Presence (n = 19)	Absence (n = 6)	P value
**Implant period (months)**	4.00 [0.38, 69.00]	89.50 [50.75, 126.00]	0.060
**Group of implant period**			0.098
<3 months	9.00 (47.37)	0.00 (0.00)	
3–6 months	1.00 (5.26)	1.00 (16.67)	
>6 months	9.00 (47.37)	5.00 (83.33)	
**Contrast uptake, n(%)**			<0.001
Without	0.00 (0.00)	6.00 (100.00)	
Partially	10.00 (52.63)	0.00 (0.00)	
Completely	9.00 (47.37)	0.00 (0.00)	
Coverage of fibrotic tissue (quadrants)*			
Left discs	1.00 [0.00, 2.00]	4.00 [4.00, 4.00]	<0.001
Right discs	0.00 [0.00, 3.50]	4.00 [4.00, 4.00]	0.004
**Complete coverage of fibrotic tissue and superposed neo-endothelia, n(%)**	0.00 (0.00)	0 .00 (0.00)	>0.999
**Complete coverage of fibrotic tissue and partial neo-endothelia, n(%)**	0.00 (0.00)	4 .00 (66.67)	0.001
**Endothelialization rate, n(%)**			
Left discs	0.00% [0.00%, 25.00%]	20.00% [2.50%, 45.00%]	0.296
Right discs	0.00% [0.00%, 29.00%]	0.00% [0.00%, 15.00%]	0.684
**Inflammation, n(%)**	2 .00 (10.53)	0.00 (0.00)	>0.999
**Calcification, n(%)**	1.00 (5.26)	0.00 (0.00)	>0.999
**Thrombus formation, n(%)**	8.00 (42.11)	0.00 (0.00)	0.129

Values are n (%), or mean ± SD.

(* Divide the disk into four quadrants centered on the hub. Quadrants covered by fibrous tissue to indicate the degree of fibrosis)

**Fig 3:**
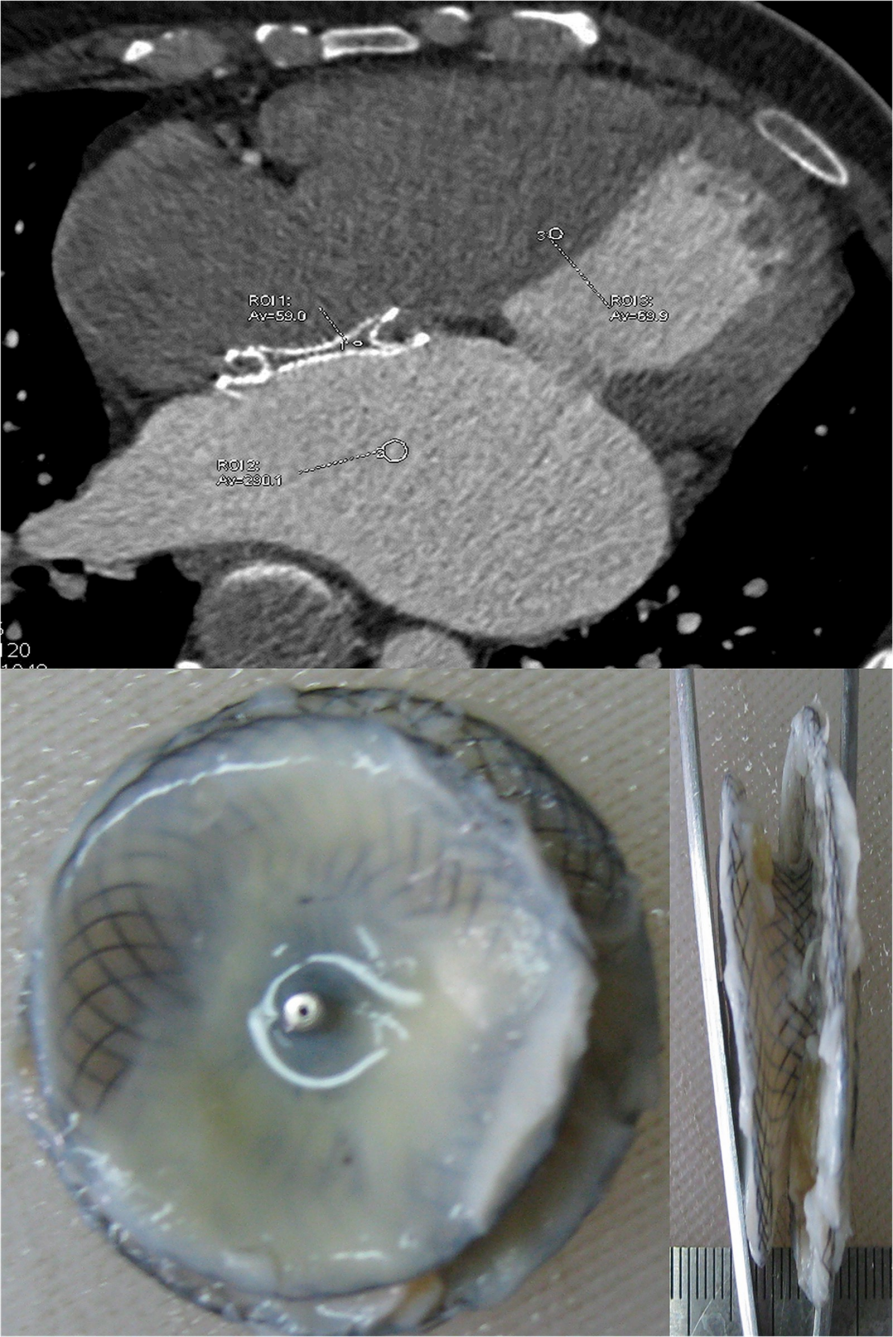
No contrast uptake within the occluder on cardiac computed tomography angiography (CTA). Cardiac CTA showed that no contrast uptake within the occluder in a patient with an implantation period of 3 years (upper panel). The local CT density within the device was lower than that of the myocardium, and the disc was completely covered by a smooth white layer of neo-endothelium (lower panel). The histological sections showed that the fibrotic tissues and superposed endothelium were identified on the completed endothelialized device. *Note*: *local CT density was 59*.*0 HU within the device*, *69*.*6 HU in the myocardium*, *and 290*.*1 HU in the left atrium; ROI*, *region of interest; Av*, *average CT density; CT*, *computed tomography; HU*, *Hounsfield units*.

On the surfaces of the devices without contrast uptake (n = 6), there was full coverage of the fibrotic tissue with an incomplete range of superposed neoendothelium in 4 patients (2 with an an atrial septal occluder, 2 with a patent ductus occluder). In addition, a smooth white layer of neoendothelium was identified along the waist of the device, which tended to grow from the peripheral area to the center. Furthermore, no thrombi were observed adjacent to the device (**Fig 3**). On the surfaces of the other 2 devices (1 ventricular septal occluder and 1 patent ductus occluder), incomplete coverage of fibrotic tissue was observed, although most of the surface was covered, with minimal superposed neoendothelium.

#### Late device endothelialization on pathology

**Fig 4:**
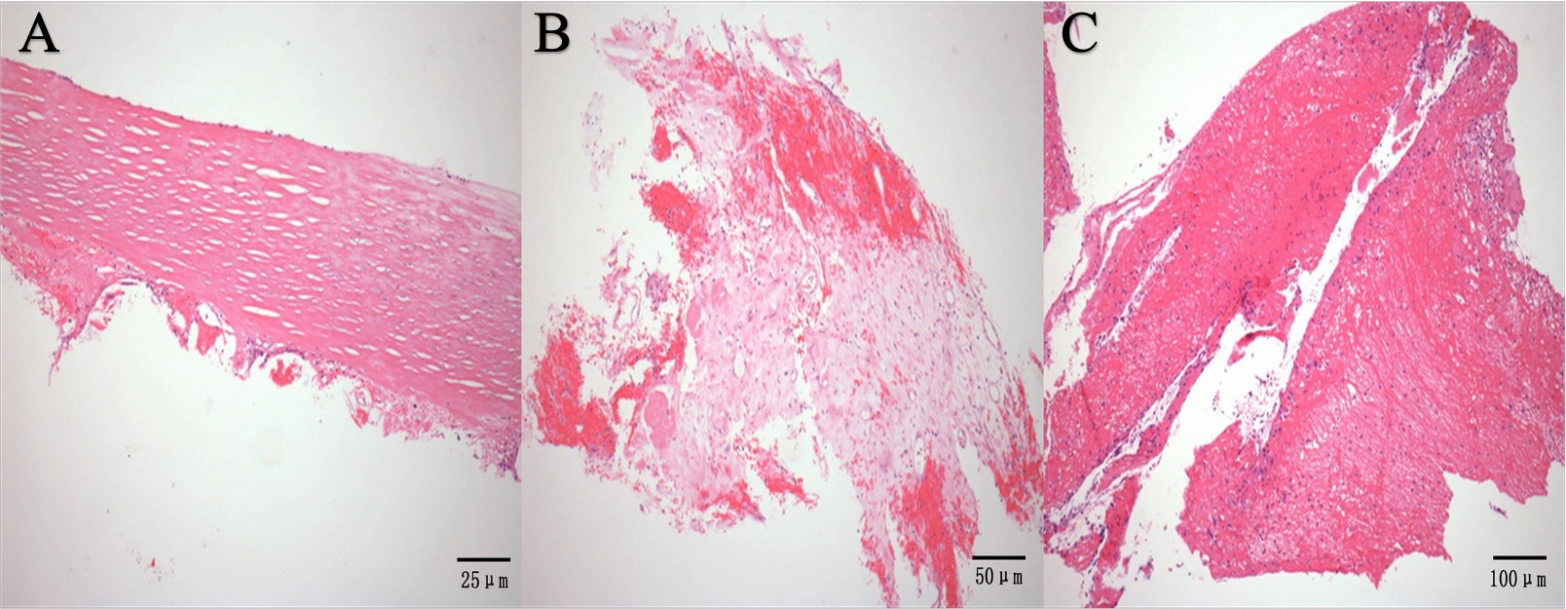
Histopathological features of tissues covered on the surface of the device. (A) Dense collagen fibers were seen on the surface of the device with a few lymphocytic cells infiltration on the proximal side of the device (H&E staining, original magnification 100×). (B) The granulation tissue and mixed thrombi were seen on the surface of the device, suggestive of organized thrombosis (H&E staining, original magnification 200×). (C) Mixed thrombus consisting of platelets, fibrin, and inflammatory cellswas seen on the surface of the device (H&E staining, original magnification 200×).

Among patients with an implantation period exceeding 6 months (n = 14), inadequate coverage of both fibrotic tissue and superposed neoendothelium occurred in 71.43% of individuals (n = 10) for the left discs of devices, and in 42.86% of patients (n = 6) under the same conditions for the right discs. Additionally, complete coverage of fibrotic tissue and incomplete superposed endothelia were identified in the left discs of 4 patients (28.57%) and in the right discs of 8 patients (57.14%), Fibrotic tissue was found histologically in the incomplete endothelialized areas, and organized thrombi were detected in 4 patients (**Table 3**). On the surface of the device, a chronic inflammatory response was observed in 2 patients and tiny calcification in 1 **(Fig 4)**. Correlation analysis demonstrated that Implantation duration was strongly associated with all pathological indices (left disc fibrosis r = 0.698, right disc r = 0.824; left disc endothelialization r = 0.598, right disc r = 0.613; all p<0.01). Antiplatelet therapy correlated with disc fibrosis (r = 0.459, p<0.05) and endothelialization (r = 0.708, p<0.01). Anticoagulation therapy was inversely correlated with endothelialization (r = -0.420, p<0.05). BMI correlated with disc fibrosis (r = 0.349) and endothelialization (r = 0.416, p<0.05). Occluder thickness showed negative correlation with right disc fibrosis (r = -0.356), while device size showed no significant correlations.(**Fig 1‘ in S1 Text).**

**Table 3:** Endothelialization of devices with implant period > 6 months or ≤ 6 months.

	Overall (N = 25)	>6 months (N = 14)	≤6 months (N = 11)	P
**Partial endothelialization, n(%)**				
Left discs	25.00 (100.00)	14.00(100.00)	11.00 (100.00)	NA
Right discs	25.00 (100.00)	14.00 (100.00)	11.00 (100.00)	NA
**Partial coverage of fibrotic tissue and partial coverage of neo-endothelia, n(%)**				
Left discs	20.00 (80.00)	10.00 (71.43)	10.00 (90.91)	0.775
Right discs	16.00 (64.00)	6.00 (42.86)	10.00 (90.91)	0.009
**Complete coverage of fibrotic tissue and partial coverage of neo-endothelia, n(%)**				
Left discs	5.00 (20.00)	4.00 (28.57)	1.00 (9.09)	0.341
Right discs	9.00 (36.00)	8.00 (57.14)	1.00 (9.09)	0.003
**Thrombus formation, n(%)**	8.00 (32.00)	4.00 (28.57)	4.00 (36.36)	>0.999
**Inflammation, n(%)**	2.00 (8.00)	2.00 (14.29)	0.00 (0.00)	0.487
**Calcification, n(%)**	1.00 (4.00)	1.00 (7.14)	0.00 (0.00)	>0.999

(Values are median n (%))

## Discussion

Cardiac CTA demonstrated potential in the evaluation of endothelialization of the device based on contrast uptake within the occluder. Our findings indicate a strong relationship between contrast uptake and incomplete endothelialization of the device, validated pathologically. Furthermore, late device endothelialization was a commonly observed histopathological feature. To the best of our knowledge, this is the first study to investigate the pathological association of cardiac CTA for the evaluation of device endothelialization in humans.

Within the occluder, contrast uptake on cardiac CTA indicated incomplete device endothelialization. The evaluation of device endothelialization through cardiac CTA varies with radiologist expertise. Experienced radiologists can make reliable visual assessments, while less experienced radiologists should rely on quantitative CT density comparison between device ROIs and myocardial tissue. Standardized training enables consistent implementation of both approaches in differentiating complete versus partial enhancement patterns. Previous studies have suggested that late device endothelialization may be associated with thrombus formation and endocarditis, which are severe postoperative complications [17–22]. Therefore, it is crucial to determine the status of device endothelialization, which determines the optimal antiplatelet/anticoagulation therapy duration until complete endothelial coverage of the implanted device [23]. In this study, cardiac CTA demonstrated potential for us to evaluate the endothelialization of devices and provide additional information to guide antiplatelet and prophylactic therapy duration after device implantation. In devices with late endothelialization, the fibrotic tissue fails to cover the surface of the occluder, which might increase the risk of thrombus formation [19, 24]. Although no thrombus-related complications were recorded, tiny thrombi were frequently detected on the polyester fibers within the device, which may become a dangerous embolic source [18, 24, 25]. Further research is required to determine the potential risks associated with these types of thrombi.

On the surface of the devices without contrast uptake, the pathological findings demonstrated complete coverage of the fibrotic tissue in two thirds of patients. In the others, the fibrotic tissue only covered the majority of the discs. Additionally, no patients had complete coverage of the neoendothelium on the device, and partial coverage occurred in only a few patients. Prior investigations have employed CTA for device endothelialization assessment through contrast enhancement pattern analysis. However, these studies were primarily observational in nature and lacked systematic histopathological validation with adequate sample sizes [12, 13]. Our findings given that the blood-contacting surfaces of devices are not biocompatible, the presence of fibrotic tissue may be a crucial step in the coverage of neonatal endothelial cells, which contributes to endothelial cell adhesion, migration, and proliferation, eventually creating an endothelial layer on the surface [26, 27]. Further research is required to determine the clinical significance of devices with complete coverage of the fibrotic tissue and an incomplete neoendothelium. This carries the potential risk of infective endocarditis, as supported by a prior pathological study that linked infective endocarditis with incomplete neointimal coverage [28]. In patients with partial contrast uptake, pathology confirmed incomplete coverage of the fibrotic tissue, and some were associated with a partial neoendothelium on the device.

In this study, late device endothelialization was observed to be common upon pathological examination. It is globally accepted that 6 months of anti-aggregation therapy is sufficient [23]. However, our findings suggest that a 6-month course may be insufficient for some patients. Among the patients with an implantation period > 6 months, significant individual variations were observed in both timing and extent of device endothelialization, most had incomplete coverage of both the fibrotic tissue and superposed endothelium in the left discs of the devices. Given the widespread acceptance and increasing global adoption of occluder devices, delayed endothelialization is expected to be encountered more frequently, necessitating extended duration of routine antiplatelet therapy, though its underlying mechanisms remain to be elucidated [9, 29–32].

Late device endothelialization is frequently detected in pathology, making it necessary to develop an imaging modality to assess endothelialization in vivo. In this study, evaluation of incomplete device endothelialization based on intra-device contrast enhancement showed high consistency with histopathological examination; furthermore, the absence of contrast uptake predominantly corresponded to pathologically verified complete endothelialization, suggesting its potential utility as a relatively effective non-invasive assessment modality. The mechanisms of late device endothelialization in humans are not fully understood, but some predictors have been proposed [33, 34]. Correlation analysis in this study demonstrated that delayed device endothelialization was significantly associated with implantation duration and antiplatelet regimen, complementing existing evidence. On the surface of devices with late endothelialization, inflammatory responses and tiny calcifications were detected, which may play an important role in late device endothelialization. The related mechanism remains unclear and might be associated with the reaction of the nickel–titanium alloy or potential graft infection.

### Study limitations

In this retrospective study, the small sample size and utilization of multiple occluder types limited the generalizability of the results. However, our study included a relatively larger number of patients compared to other single-center studies. Despite variations in occluder types, their design structures remained consistent, all crafted from nitinol alloy. Additionally, occluder removal necessitated by other cardiovascular diseases or device embolization represents a potential limitation. Although this might introduce selection bias, pre-procedural CTA evaluation was exclusively performed in patients meeting the inclusion criteria, and these cardiovascular conditions did not compromise the validity of CTA assessment.

### Conclusions

On cardiac CTA, contrast uptake within the occluder indicated incomplete device endothelialization with a satisfactory pathological correlation. Furthermore, late device endothelialization is not uncommon, and further research is required to determine its underlying mechanism.

## Supporting information

S1 Text Supplementary materials for occluder device endothelialization study. This supplementary information contains comprehensive data including tabulated baseline characteristics of various occluder devices(Table A), quantitative correlation analyses of factors influencing tissue fibrosis and endothelialization processes (Fig 1‘), scanning electron microscopic characterization of fibrous tissue formation and endothelial cell morphology (Figs 2, 3), and clinical imaging documentation of device-related procedures including surgical explantation of left atrial appendage occlude (Fig 4‘), patent ductus arteriosus (PDA) occluder removal (Fig 5’), and contrast-enhanced imaging of atrial septal occluder integration (Fig 6’).
(DOCX)

## Abbreviations

CTA: Computed tomography angiography
ROI: Region of interest
HU: Hounsfield units
