# Supplementary material for: Evaluation of endothelialization of an occluder device with cardiac computed tomography and assessment of the pathological validation
Source: PLoS One. 2025 Jan 10;20(1):e0316638. doi: 10.1371/journal.pone.0316638 (PMC11723591; doi:10.1371/journal.pone.0316638)
Supplement: S1 Text — This supplementary information contains comprehensive data including tabulated baseline characteristics of various occluder devices(Table A), quantitative correlation analyses of factors influencing tissue fibrosis and endothelialization processes (Fig 1‘), scanning electron microscopic characterization of fibrous tissue formation and endothelial cell morphology (Figs 2, 3), and clinical imaging documentation of device-related procedures including surgical explantation of left atrial appendage occlude (Fig 4‘), patent ductus arteriosus (PDA) occluder removal (Fig 5’), and contrast-enhanced imaging of atrial septal occluder integration (Fig 6’). (DOCX) [file pone.0316638.s001.docx]

**Supporting information**

**S1 TEXT. Supplementary Materials for Occluder Device Endothelialization Study.** This supplementary information contains comprehensive data including tabulated baseline characteristics of various occluder devices(Table A), quantitative correlation analyses of factors influencing tissue fibrosis and endothelialization processes(Fig 1’), scanning electron microscopic characterization of fibrous tissue formation and endothelial cell morphology(Fig2’-3’), and clinical imaging documentation of device-related procedures including surgical explantation of left atrial appendage occlude(Fig 4’), patent ductus arteriosus (PDA) occluder removal(Fig 5’), and contrast-enhanced imaging of atrial septal occluder integration(Fig 6’).

**Table A. Baseline Characteristics of the Different Device Types**

|  | | **Patients** | | **Device Type** | | |
| --- | --- | --- | --- | --- | --- | --- |
|  | | **Overall （N=25)** | | **ASO (N=16)** | **Others（N=9)** | **P** |
| **Age (yrs)** | | 50.00 [17.00, 52.00] | | 50.00 [24.50, 51.25] | 49.00 [14.00, 56.00] | 0.865 |
| **Female, n(%)** | | 12 (48.00) | | 10 (62.50) | 2 (22.20) | 0.097 |
| **Height (cm)** | 165.00 [155.00, 170.00] | | 163.50[154.50,169.25] | | 170.00[163.00,174.00] | 0.192 |
| **Weight (kg))** | | 56.84 ± 18.06 | | 56.31 ± 14.36 | 57.78 ± 24.30 | 0.850 |
| **BMI (kg/** **m2)** | | 21.71 ± 4.08 | | 22.00 ± 3.79 | 21.20 ± 4.76 | 0.651 |
| **Device size (mm)** | | 19.56 ± 9.74 | | 23.42 ± 7.69 | 12.69 ± 9.53 | 0.005 |
| **Device thickness (mm)** | | 12.02 ± 3.77 | | 12.42 ± 3.01 | 11.32 ± 4.97 | 0.497 |
| **Antiplatelet period (months)** | | 0.50 [0.00, 6.00] | | 6.00 [0.50, 6.00] | 0.00 [0.00, 0.00] | 0.004 |
| **Implant period（months）** | | 54.00 ± 62.97 | | 58.45 ± 64.01 | 46.09 ± 64.05 | 0.134 |

**Values are median [IQR] (Interquartile range), n (%), or mean ± SD.**

**(BMI=body mass index; ASO = atrial septal occluder)**

**Fig 1’:** Correlation Analysis of Factors Associated with Fibrosis and Endothelialization Rates.


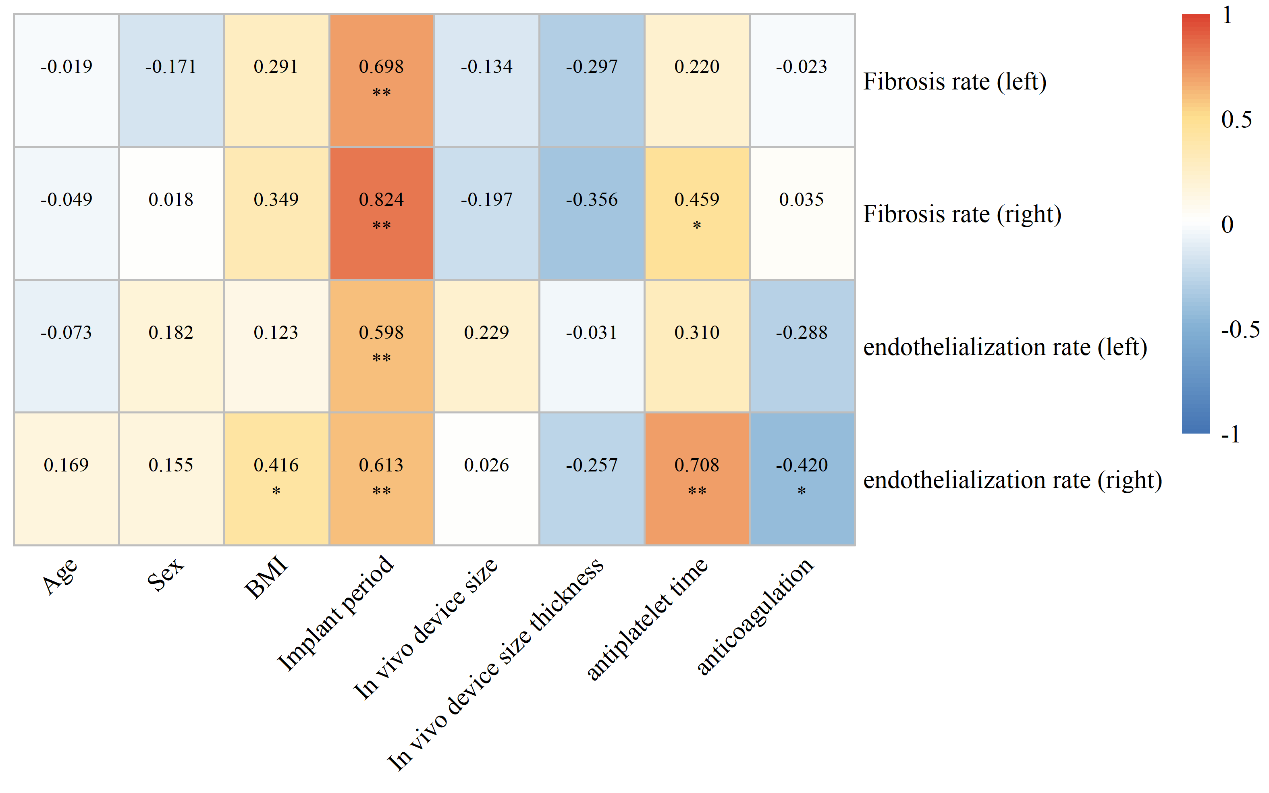


**Legend:** Spearman correlation analysis of each influencing factor with fibrosis and endothelialization, the numbers in the figure indicate the magnitude of correlation (ρ), and ρ ranges from -1 to 1, with closer to 1 or -1 indicating stronger correlation. ρ<0 shows a negative correlation, and ρ>0 indicates a positive correlation. In the graph, * displays P<0.05, and ** indicates P<0.01. The analysis results showed that the Implant period showed moderate to strong positive correlation with all four indexes, antiplatelet time was moderately positively correlated with Fibrosis rate (right) and endothelialization rate (right), Anticoagulation was moderately positively correlated with Anticoagulation was moderately negatively correlated with endothelialization rate (right), and BMI was moderately positively correlated with endothelialization rate (right). Therefore, BMI, implant period, antiplatelet time, and anticoagulation are the most critical factors in fibrosis; anticoagulation is a potential influence on fibrosis and endothelialization.

**Fig 2’ :** Electron Microscopic Images of Fibrous Tissue.


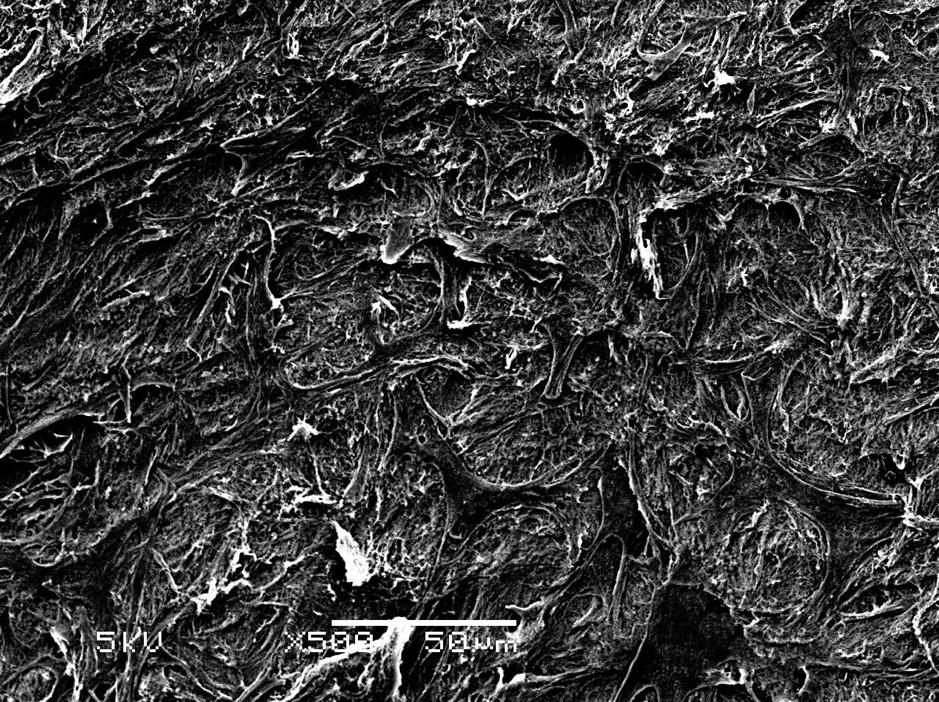


**Legend:** The samples were fixed, dehydrated, embedded, dried and vacuum ion-sprayed, and then observed under JSM5050 scanning electron microscope. Fibrous tissue formations, devoid of endothelial cell presence (SEM, original magnification 500×). Note: JSM5050, Japan-made electron microscope model.

**Fig 3’ :** Electron Microscopic Imaging of Endothelial Cells.


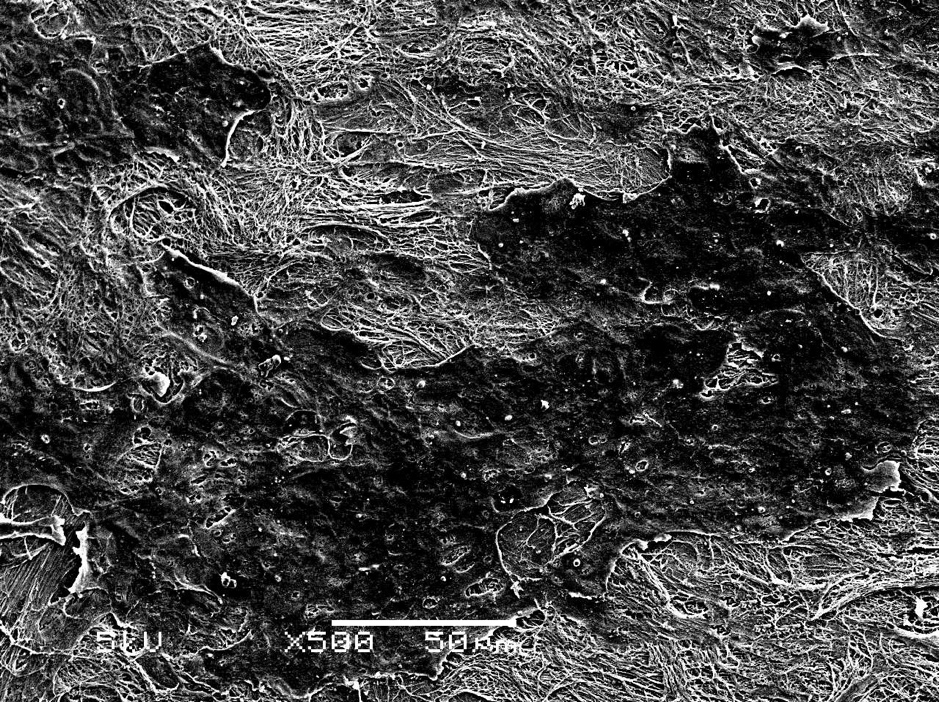


**Legend:** The specimens were fixed, dehydrated, embedded, dried and vacuum ion-sprayed, and then observed under JSM5050 scanning electron microscope. Fibrous tissue andfocal endothelial cell formation can be seen (SEM, original magnification 500×).

**Fig 4’:** Surgical Explantation of Left Atrial Appendage Occluder


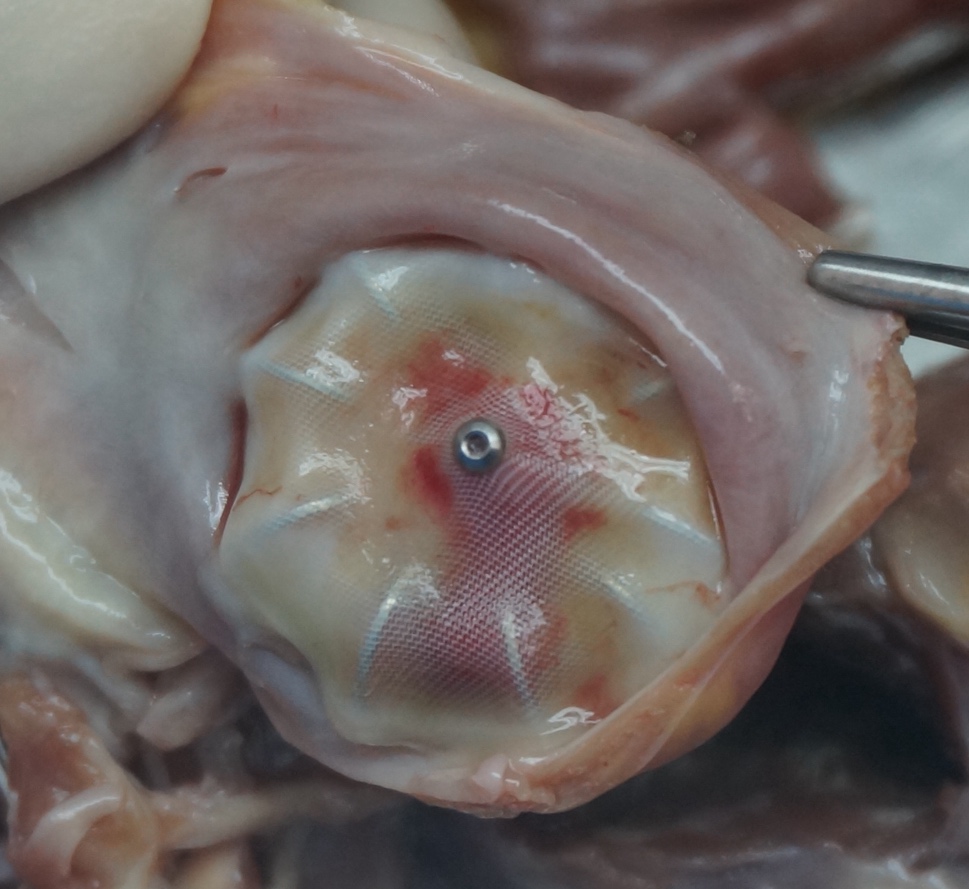


**Legend:** An overview of the atrial appendage occluder after surgical removal, showing the device surface entirely covered by grey-white fibrous tissue *Note：LAA，left atrial appendage*.

**Fig 5’ :**Surgical removal of PDA occulder


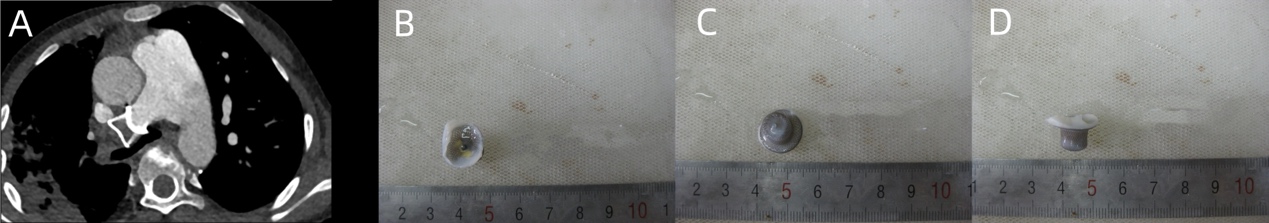


**Legend:** (A): Cardiac CTA revealed that the occluder with no contrast uptake, then surgical removal due to occulder displacement. (B): In frontal view, the surface of the occluder was completely covered with a relatively dense layer of milky white biofilm-like tissue. (C): A thin layer of white biofilm tissue was seen on the other side of the occlude. (D): In lateral view, a little white tissue wrapping was visible at the waist of the occluder. *Note:* *PDA , patent ductus arteriosus; CTA, computed tomography angiography.*

**Fig 6’:** Partial contrast uptake within the atrial septal occluder

**
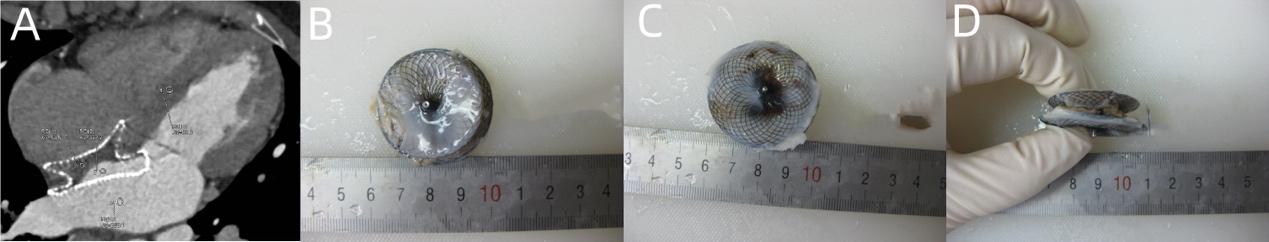
**

**Legend:** (A): Cardiac CTA show partial contrast uptake in the left disk of the septal occluder and no contrast uptake in the right disk. (B): Dense milky white membranous tissue covered the surface of the right disk of the occlude. (C): Occluder partial coverage of white biofilm-like tissue on the left disk. (D): Lateral view of the occluder with a small amount of white tissue wrapped around the lumbar area. *Note: local CT density was 377.7 HU within the contrast uptake area of the device,63.5 HU within the no contrast uptake area, 83.3 HU in the myocardium, and 393.1 HU in the left atrium; ROI, region of interest; Av, average CT density; CT, computed tomography; HU, Hounsfield units.* *CTA, computed tomography angiography.*
